# Population-based prevalence of cervical infection with human papillomavirus genotypes 16 and 18 and other high risk types in Tlaxcala, Mexico

**DOI:** 10.1186/s12879-016-1782-x

**Published:** 2016-09-01

**Authors:** Samantha E. Rudolph, Attila Lorincz, Cosette M. Wheeler, Patti Gravitt, Eduardo Lazcano-Ponce, Leticia Torres-Ibarra, Leith León-Maldonado, Paula Ramírez, Berenice Rivera, Rubí Hernández, Eduardo L. Franco, Jack Cuzick, Pablo Méndez-Hernández, Jorge Salmerón, FRIDA Study Group, Samantha E. Rudolph, Attila Lorincz, Cosette Wheeler, Patti Gravitt, Eduardo Lazcano, Leticia Torres-Ibarra, Leith León, Paula Ramírez, Berenice Rivera, Eduardo L. Franco, Jack Cuzick, Pablo Méndez, Jorge Salmerón, Mauricio Hernández, Thomas C. Wright, Anna Barbara Moscicki, Yvonne Flores, Mark H. Stoler, Enrique Carmona, Kathleen M. Schmeler, David Bishai, Pilar Hernández, Daniel Alvarez, Elizabeth Barrios, Rubi Hernández, Indira Mendiola, Vicente González

**Affiliations:** 1 UC Berkeley-UCSF Joint Medical Program, Berkeley, CA USA; 2 Unidad de Investigación Epidemiológica y en Servicios de Salud, Instituto Mexicano del Seguro Social, Cuernavaca, Morelos, México; 3 Wolfson Institute of Preventive Medicine, Barts and The London School of Medicine, Queen Mary University of London, London, UK; 4 Department of Pathology, University of New Mexico School of Medicine, Albuquerque, NM USA; 5 Centro de Investigación en Salud Poblacional, Instituto Nacional de Salud Pública, Avenida Universidad 655, Colonia Sta. María Ahuacatitlán, 62100 Cuernavaca, Morelos Mexico; 6 CONACYT, Centro de Investigación en Salud Poblacional, Instituto Nacional de Salud Pública, Cuernavaca, Morelos México; 7 Division of Cancer Epidemiology, McGill University, Montreal, Canada; 8 Departamento de Enseñanza, Capacitación e Investigación, Secretaría de Salud de Tlaxcala, Tlaxcala, Tlaxcala Mexico; 9 Facultad de Ciencias de la Salud, Universidad Autónoma de Tlaxcala, Tlaxcala, Tlaxcala Mexico

**Keywords:** Human papillomavirus DNA testing, HPV16/18, Prevalence, Risk factors, Mexico

## Abstract

**Background:**

Cervical cancer remains an important cause of cancer mortality for Mexican women. HPV 16/18 typing may help to improve cervical cancer screening. Here we present the prevalence of high-risk human papillomavirus (hrHPV) including HPV16 and HPV18 from the FRIDA (Forwarding Research for Improved Detection and Access) population.

**Methods:**

Beginning in 2013, we recruited 30,829 women aged 30–64 in Tlaxcala, Mexico. Cervical samples were collected and tested for 14 hrHPV genotypes (16, 18, 31, 33, 35, 39, 45, 51, 52, 56, 58, 59, 66, and 68). We used logistic regression to estimate odds ratios with 95 % confidence intervals for hrHPV infections according to putative risk factors.

**Results:**

Prevalence of infection with any of the 14 hrHPV types was 11.0 %. The age-specific prevalence of all hrHPV formed a U-shaped curve with a higher prevalence for women aged 30–39 and 50–64 than women aged 40–49. Across all age groups, 2.0 % of women were positive for HPV16 and/or HPV18 (HPV16/18), respectively. HPV16/18 prevalence also showed a U-shaped curve with increased prevalence estimates for women aged both 30–39 and 60–64. Both prevalence curves had a significant quadratic age coefficient. Infections with hrHPV were positively associated with an increased number of lifetime sexual partners, a history of sexually transmitted disease, being unmarried, use of hormonal contraception, having a history of smoking and reported condom use in the multivariate model.

**Conclusions:**

The FRIDA population has a bimodal distribution of both hrHPV and HPV16/18 positivity with higher prevalences at ages 30–39 and 60–64. These findings will help to evaluate triage algorithms based on HPV genotyping.

**Trial registration:**

The trial is registered with ClinicalTrials.gov, number NCT02510027.

## Background

Cervical cancer is the second most fatal cancer for women in Mexico [1]. Chronic infection with high-risk human papillomavirus (hrHPV), one of the world’s most prevalent sexually transmitted infections (STIs), is a necessary cause of cervical cancer [2]. Over 100 types of HPV have been established. Among these, 15 HPV types are classified as carcinogenic or high risk (16, 18, 31, 33, 35, 39, 45, 51, 52, 56, 58, 59, 68, 73, and 82) [3]. From the point of view of cervical pathology, however, two types—HPV16 and 18—account for the vast majority of cervical cancer cases. Worldwide, HPV16 alone accounts for almost 60 % of cases, and HPV18 accounts for another 10 % of cases [4].

Because many countries in the developing world including Mexico do not have reliable cancer registries, it is difficult to ascertain the incidence of disease. Without incidence rates, resource allocation cannot fully reflect the differing needs of the various health districts. The prevalence of hrHPV, particularly HPV16 and HPV18, can be used as a valuable predictor of cervical cancer incidence [5].

Wise resource allocation will become more important as the cervical cancer prevention landscape in Mexico shifts. In 2008, Mexico began using hrHPV testing instead of cervical cytology for primary screening in the public sector, and then in 2012, Mexico introduced a universal HPV vaccination program for all girls in the 5^th^ grade of elementary school [6]. The impact of the vaccine, however, will not be seen for at least a generation, and therefore, research groups in Mexico and throughout the developing world are seeking to establish their cervical cancer screening program. At the current time, Mexican health services cannot support a screening program in which all hrHPV positive women are sent for further diagnostic work-up [7]. Indeed only a small percentage of hrHPV positive women will actually ever develop high grade cervical intraepithelial neoplasia (CIN) [8].

To avoid overburdening the local services, researchers are now working to identify a reliable triage testing strategy for hrHPV positive women. While cytology seems to be an obvious triage option given the existing infrastructure, we are also trying alternatives because of the difficulty of maintaining high-quality cytology in Mexico [9]. One potentially important option for the triage of hrHPV positive women would be to identify those positive for HPV16 and HPV18 for further evaluation and diagnostic confirmation. Results of the Roche ATHENA trial in the US demonstrate the strong promise of HPV16/18 based triage strategies to increase diagnostic efficiency without sacrificing sensitivity of disease detection [10]. Indeed Roche has recently received FDA approval for its test to triage HPV16/18 positive women for diagnostic follow-up. The feasibility of a similar screening strategy in Mexico will rest on the prevalence of HPV16 and HPV18 and the positive predictive value of these types for high grade CIN.

The prevalence of HPV16 and HPV18 within the general population has not been well described in Mexico. The last population-based study to report on the prevalence of HPV16 and HPV18 in Mexico was published over a decade ago in 2001 [11]. More recent studies have only looked at small convenience samples [12–14]. The present study is part of a larger study, FRIDA (Forwarding Research for Improved Detection and Access), a population-based demonstration project designed to evaluate the performance and cost-effectiveness of triage alternatives for cervical cancer screening in Tlaxcala, Mexico. While a number of small studies have reported on the type-specific prevalence of HPV16 and HPV18 in Mexico [11–18], the present study is the largest to do so with population-based representation.

## Methods

### Study population

**Fig. 1:**
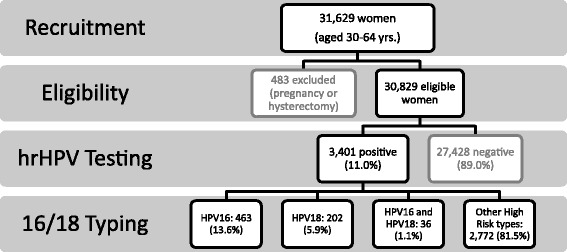
Flow Chart of hrHPV Screening of the FRIDA Study Population. Women 30 to 64 years of age living within our target health district were invited by healthcare personnel. This study reports the results from the first 31,629 women who volunteered to participate in the Tlaxcala cervical cancer screening program. Four-hundred and eighty-three women were excluded, leaving 30,829 who had hrHPV results available, in the current analysis. Of those 30,829 women, 3,401 women were positive for hrHPV. Among those 3,401 women, 13.6 % were positive for HPV16, 5.9 % for HPV18 and 1.1 % for both HPV16 and HPV18 coinfection. These three categories indicate positivity independent of the presence of other hrHPV types. The last category of other high risk HPV types include women who tested positive for other hrHPV types (31, 33, 35, 39, 45, 51, 52, 56, 58, 59, 66, and 68), but neither HPV16 nor HPV18

We studied specimens and data from the FRIDA study, an on-going population-based demonstration project that began in Tlaxcala, Mexico in August 2013. Women 30 to 64 years of age living within our target health district in Tlaxcala, Mexico are being recruited directly by healthcare personnel within preventive, family planning and gynecological health services during routine visits. This target health district includes approximately 100 health care facilities. The complete FRIDA cohort is projected to include more than 80% of the target population of over 100,000 women aged 30 to 64 years. Although the Tlaxcala cervical cancer screening program continuously invites women for screening at regular time intervals, women are generally tested opportunistically, with screening usually being initiated by the woman herself. This preliminary analysis includes results from the first 31,629 women enrolled in the demonstration project who came to the the clinic health care settings. Women then were invited at the clinic into the study. Women who were pregnant or had had a hysterectomy were excluded, leaving a total of 30,829 women in the present analysis (Fig. 1).

The study proposal was reviewed and approved by the research and ethics committees of the Instituto Nacional de Salud Pública (INSP) (1094), Comisión Federal para la Protección Contra Riesgos Sanitarios (CAS/OR/01/CAS/123300410C0044-3578/2012) and Secretaría de Salud del Estado de Tlaxcala (SS. DECI-OI-13/12). The purpose and procedures of the study were explained to all participants and informed verbal consent was obtained.

### Procedures

Health care personnel conducted face-to-face interviews of FRIDA study participants using pre-printed pen and paper surveys designed to elicit details of sexual behavior, history of STIs, parity, gynecologic and reproductive health histories, and other probable risk factors for cervical cancer.

Participants were then given a pelvic exam during which a cervical sample was collected using a Cervex-Brush® (Rovers®). This cervical sample was placed in a ThinPrep® vial (Hologic, Inc., Bedford, MA) and then 0.4 mL tested for hrHPV using Roche’s cobas® 4800 system. This assay uses a PCR-based in-vitro test for the simultaneous detection of 14 hrHPV types (16, 18, 31, 33, 35, 39, 45, 51, 52, 56, 58, 59, 66, and 68) in liquid based cervical samples. The system detects HPV16 and HPV18 individually, and the remaining 12 hrHPV genotypes as a pool [19]. The testing was carried out at the HPV laboratory at the INSP in Cuernavaca, Mexico according to manufacturer’s instructions (Roche Diagnostics).

### Statistical analysis

Descriptive statistics were used to characterize the variables. Age, age of sexual debut, parity, and number of sexual partners were recorded as continuous, numeric variables, and then analyzed as categorical variables. Marital status, use of contraception (hormonal, IUD, condoms), history of previous STIs, and smoking history were both collected and analyzed as categorical variables. In the analysis, participants classified as hrHPV positive tested positive for any of the 14 hrHPV types including HPV16 and HPV18. Among these positives, participants were then classified as positive for HPV16 regardless of whether or not they also tested positive for another hrHPV type. Participants classified as positive for HPV18 were treated in the same way. The designation “HPV16/18” refers to participants who tested positive for either HPV16, HPV18, or both, regardless of other hrHPV types. The designation “HPV16/18 only” means these participants tested positive for HPV16 and/or HPV18, but no other hrHPV types. The “non-16/18 hrHPV only” group refers to women who tested positive for other hrHPV types (31, 33, 35, 39, 45, 51, 52, 56, 58, 59, 66, and 68), but neither HPV16 nor HPV18. The “HPV16/18 and other hrHPV types” group tested positive for HPV16 and/or HPV18 as well as another hrHPV type.

All pooled hrHPV, HPV16/18 only, non-16/18 hrHPV only, and HPV16/18 and other hrHPV type prevalence were estimated by age along with associated binomial 95 % confidence intervals (CI). Looking at the prevalence graphs by age categories, we decided to use a quadratic model for log odds as a function of age. Assuming that the quadratic models are approximately correct, the coefficient on age^2^ is a rough measure of the steepness of the parabola. A formal test for differences in steepness of curves for different hrHPV categories was constructed by using the coefficient estimates and their standard errors. Because the sample sizes are large, a normal approximation was used.

We furthermore used logistic regression to compute odds ratios (ORs) with 95 % CIs to identify independent risk factors for hrHPV infections. ORs were adjusted for age to account for differences in age-group prevalence and for comparability with other population reports. Variables associated with hrHPV infections were adjusted for other covariates using multiple logistic regression analysis. Data analysis was conducted using STATA version 12.0 (StataCorp). A two-sided significance level of 0.05 was chosen to indicate statistical significance.

## Results

### Characteristics of the population

Nearly 97% of women who volunteered to participate in the Tlaxcala cervical cancer screening program met the inclusion study criteria. Approximately 31 % of the target population is included in this analysis. The distribution of ages screened does not perfectly reflect the target population. A higher proportion of women aged 30 to 49 were screened than older groups in the population. Approximately 3.4 % of women aged 60 to 64 were screened. The mean age of the study population was 41.8 years (data not shown).

Approximately 90 % of participants were married, and nearly two-thirds of participants were 18 or older when they initiated sexual activity. The majority of participants have been pregnant and almost 50 % report 3 or more live births. The vast majority of the study participants reported one lifetime sexual partner (71 %). A history of oral contraceptive use was reported by about 21 % of women and a little more than one-third (33 %) were using an IUD. Most women (83 %) reported not using a condom at all in the last 12 months. Histories of previous sexually transmitted infections were rare (3 %), and about 3 % of women reported ever having smoked (Table 1). Approximately 5.0 % self-reported a history of abnormal cervical cytology (data not shown) and 24.9 % of those women received treatment.

**Table 1:** Associations between hrHPV and Reproductive Health and Behavior Variables

	n (% of Total pop. screened)	hrHPV n	Prevalence hrHPV (95 % CI)	Age Adjusted OR (95 % CI)^a^	Multivariate Adjusted OR (95 % CI)^b^
Age (years)					
30–34	7,083 (23.0)	971	13.7 (12.9–14.5)	**1.65 (1.46–1.87)**	**1.61 (1.42–1.83)**
35–39	7,131 (23.1)	809	11.3 (10.6–12.1)	**1.33 (1.17–1.51)**	**1.31 (1.15–1.49)**
40–44	6,176 (20.0)	582	9.4 (8.7–10.2)	1.08 (0.95–1.24)	1.09 (0.95–1.25)
45–49	4,402 (14.3)	386	8.8 (7.9–9.6)	1 (ref)	1 (ref)
50–54	2,969 (9.6)	304	10.2 (9.2–11.4)	1.19 (1.01–1.39)	1.19 (1.01–1.40)
55–59	2,015 (6.5)	230	11.4 (10.1–12.9)	**1.34 (1.28–1.59)**	**1.32 (1.11–1.58)**
60–64	1,053 (3.4)	119	11.3 (9.5–13.4)	**1.33 (1.07–1.65)**	**1.34 (1.07–1.68)**
Total	30,829	3401	11.0 (10.7–11.4)		
Marital Status					
Unmarried	3, 362 (10.9)	600	17.9 (16.6–19.2)	**1.96 (1.78–2.16)**	**1.65 (1.48–1.83)**
Married/Civil Union	27,467 (89.1)	2,801	10.2 (9.8–10.6)	1 (ref)	1 (ref)
Age of Sexual Debut					
≤17	11,221 (36.4)	1,333	11.9 (11.3–12.5)	**1.13 (1.05–1.20)**	1.04 (0.97–1.13)
≥18	19,591 (63.6)	2,067	10.6 (10.1–11.0)	1 (ref)	1 (ref)
Number of Lifetime Sexual Partners^c^					
1 partner	21,832 (70.8)	1,950	8.9 (8.6–9.3)	1 (ref)	1 (ref)
2 partners	5,910 (19.2)	879	14.9 (14.0–15.8)	**1.77 (1.62–1.93)**	**1.61 (1.47–1.76)**
≥3 partners	2,940 (9.5)	556	18.9 (17.5–20.4)	**2.34 (2.11–2.59)**	**1.96 (1.75–2.19)**
Number of Sexual Partners in the Last 12 Months^c^					
0 partners	5,724 (18.6)	686	12.0 (11.2–12.9)	1 (ref)	1 (ref)
1 partner	24,792 (78.2)	2,659	10.7 (10.3–11.1)	0.87 (0.79–0.95)	0.96 (0.87–1.05)
≥2 partners	111 (0.4)	32	28.8 (21.2–37.9)	**2.9 (1.90–4.41)**	**1.75 (1.13–2.70)**
Parity					
0	7,091 (23.1)	785	11.1 (10.4–11.8)	1 (ref)	
1 or 2	8,511 (27.6)	967	11.4 (10.7–12.1)	1.04 (0.94–1.15)	
3 or more	15,049 (48.8)	1,631	10.8 (10.4–11.3)	1.05 (0.96–1.16)	
History of Hormonal Contraception					
Yes	6,417 (20.8)	790	12.3 (11.5–13.1)	**1.17 (1.07–1.27)**	1.10 (1.01–1.20)
No	24,154 (78.1)	2,587	10.7 (10.3–11.1)	1 (ref)	1 (ref)
Current IUD Use					
Yes	10,170 (33.0)	1,164	11.5 (10.8–12.1)	1.05 (0.98–1.14)	
No	19,887 (64.5)	2,153	10.8 (10.4–11.3)	1 (ref)	
Condom Use in the last 12 months					
Never	25,557 (82.9)	2,682	10.5 (10.1–10.9)	1 (ref)	1 (ref)
Almost always	2,777 (9.0)	373	13.4 (12.2–14.8)	**1.27 (1.13–1.43)**	1.15 (1.01–1.29)
Always	1,716 (5.6)	253	14.7 (13.1–16.5)	**1.42 (1.23–1.63)**	**1.26 (1.09–1.46)**
History of a Previous STI					
Yes	979 (3.2)	182	18.6 (16.3–21.1)	**1.91 (1.62–2.25)**	**1.54 (1.30–1.84)**
No	29,424 (95.4)	3,161	10.7 (10.4–11.1)	1 (ref)	1 (ref)
Tobacco Use					
Never	29,238 (94.8)	3,160	10.8 (10.5–11.2)	1 (ref)	1 (ref)
Ever	964 (3.1)	175	18.2 (15.8–20.7)	**1.77 (1.49–2.10)**	**1.23 (1.03–1.47)**

*Abbreviations*: *STI* sexually transmitted infections, *OR* odds ratio, *CI* confidence interval

^a^Odds ratios for all the variables with the exception of age itself are adjusted for age. Age was included in the model as a categorical variable (30–34, 35–39, 40–44, 45–49, 50–54, 55–59, 60–64); ^b^Association test was adjusted for age, marital status, age of sexual debut, number of lifetime sexual partners, number of sexual partners in the last 12 months, history of hormonal contraception, condom use in the last 12 months, history of a previous STI, and tobacco use; ^c^Women who reported zero lifetime sexual partners were excluded

Statistically significant *p* values ≤ 0.05 of the OR's are marked in bold font

### High risk HPV prevalence and risk profile

**Fig. 2:**
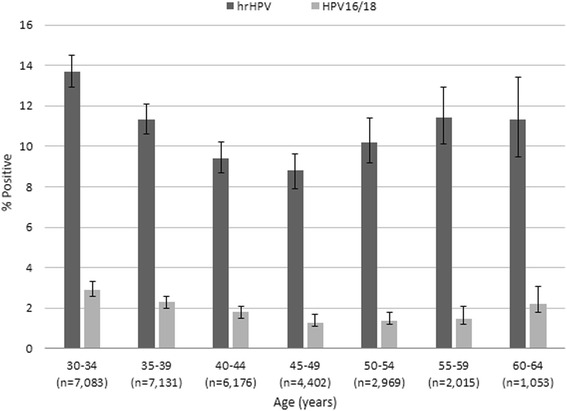
Age Specific Prevalence of hrHPV and HPV16/18 with 95 % CI. The overall prevalence of hrHPV was 11.0 % in this population. Two percent of women overall were positive for HPV16 and/or HPV18 (HPV16/18). The prevalence of hrHPV overall and HPV16/18 by age group both show a bimodal distribution with an increased prevalence for the youngest women in the population aged 30–39 and a second bump of positivity for the oldest women aged 60 and above

The estimated overall prevalence of hrHPV in this population was 11.0 % (95 % CI 10.7–11.4). High risk HPV DNA was detected in 13.7 % (95 % CI 12.9–14.5) of women aged 30 to 34 and in 11.3 % (95 % CI 10.6–12.1) of women aged 35 to 39. The prevalence estimates declined to 9.4 % (95 % CI 8.7–10.2), 8.8 % (95 % CI 7.9–9.6), and 10.2 % (95 % CI 9.2–11.4) for women aged 40–44, 45–49, and 50–54, respectively. High risk HPV detection then increased again to 11.4 % (95 % CI 10.1–12.9) and 11.3 % (95 % CI 9.5–13.4) for women aged 55 to 59 and 60 to 64, respectively (Table 1 and Fig. 2). In the multivariate model, women aged 30 to 39 were at higher risk of hrHPV infection than women aged 45 to 49, the reference group. The prevalence of hrHPV infections for the oldest age group, women aged 55 to 64, was also significantly different than the reference group, showing a clear U-shaped curve. The coefficients on age^2^ were significant.

In addition to age related risks, being unmarried, having a history of oral contraceptive use, having a history of smoking, condom use, having more than one lifetime sexual partner, having two or more sexual partners in the last year, and a history of another STI were also found to be positively associated with hrHPV infections in the multivariate model (p < 0.05). This model adjusted for marital status, age at initiation of sexual intercourse, number of lifetime sexual partners, number of sexual partners in the last 12 months, history of hormonal contraception, condom use in the last 12 months, a history of a previous STI, and tobacco use. Age of sexual initiation was, significant only in the age adjusted model (Table 1).

### Prevalence of hrHPV Infections by Type in the total screened population

**Fig. 3:**
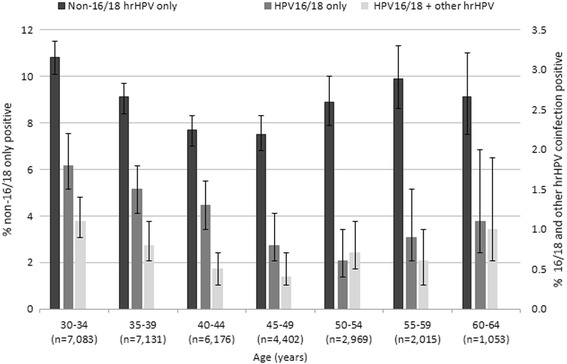
Age Specific Prevalence of hrHPV Types with 95 % CI. The cobas® 4800 system delivers hrHPV results in three categories: HPV16, HPV18, and other hrHPV. Based on these results, we divided the population into three mutually exclusive categories: (1) women positive for only other hrHPV (non-16/18 hrHPV only), (2) women positive for HPV16 and/or HPV18 (HPV16/18), and (3) women positive for HPV16 and/18 as well as another hrHPV (HPV16/18 + other hrHPV). The prevalence of these three categories by age group shows a similar bimodal distribution with increased prevalence values for the youngest and oldest women in the population

Among all age groups, 9.0 % tested positive for non-16/18 hrHPV only. The distribution by age group showed a U-shaped curve with increased prevalence estimates for women in their 30s and as well as for women over the age of 50 (Table 2 and Fig. 3). The coefficients on age^2^ were significant (Table 3).

**Table 2:** Type Specific Prevalence of hrHPV by Age

Age (years)	Total pop.	HPV16/18^a^	Non-16/18 hrHPV only	HPV16/18 only	HPV16/18 and
					Non-16/18 hrHPV
		% (95 % CI)	% (95 % CI)	% (95 % CI)	% (95 % CI)
30–34	7,083	2.9 (2.5–3.3)	10.8 (10.1–11.5)	1.8 (1.5–2.2)	1.1 (0.9–1.4)
35–39	7,131	2.3 (2.0–2.7)	9.1 (8.4–9.7)	1.5 (1.2–1.8)	0.8 (0.6–1.1)
40–44	6,176	1.8 (1.5–2.1)	7.7 (7.0–8.3)	1.3 (1.0–1.6)	0.5 (0.3–0.7)
45–49	4,402	1.3 (1.0–1.6)	7.5 (6.8–8.3)	0.8 (0.6–1.2)	0.4 (0.3–0.7)
50–54	2,969	1.4 (1.0–1.8)	8.9 (7.9–10.0)	0.6 (0.4–1.0)	0.7 (0.5–1.1)
55–59	2,015	1.5 (1.1–2.2)	9.9 (8.6–11.3)	0.9 (0.6–1.5)	0.6 (0.3–1.0)
60–64	1053	2.2 (1.5–3.3)	9.1 (7.5–11.0)	1.1 (0.7–2.0)	1.0 (0.6–1.9)
Total	30,829	2.0 (1.9–2.2)	9.0 (8.7–9.3)	1.3 (1.2–1.4)	0.7 (0.6–0.8)

*Abbreviations*: *CI* confidence interval

^a^HPV16/18 refers to women who are positive for HPV16 and/or 18 regardless of other hrHPV types for which they may or may not test positiv

**Table 3:** Quadratic Model for Log Odds of hrHPV and hrHPV by Type

	Coef. on age^2^ (SD)	Coef. on age (SD)	Model *p* value	Two-tailed P values for Differences in Coefficient on Age^2^
Any hrHPV	0.00178 (0.000299)	−0.1702 (0.0204)	<0.001	
HPV16/18^a^	0.00228 (0.000520)	−0.2302 (0.0458)	<0.001	0.206^b^
Non-16/18 hrHPV only	0.00155 (0.000250)	−0.1449 (0.0223)	<0.001	
HPV16/18 only	0.00147 (0.000681)	−0.1628 (0.0594)	0.031	1.098^c^
HPV16/18 and other hrHPV	0.00342 (0.000805)	−0.3246 (0.0717)	<0.001	

*Abbreviation*: *SD* standard deviation

^a^HPV16/18 refers to women who are positive for HPV16 and/or 18 regardless of other hrHPV types for which they may or may not test positive; ^b^comparison non-16/18 hrHPV only vs HPV16/18; ^c^ comparison non-16/18 hrHPV only vs HPV16/18 only

Among all age groups, 1.5 and 0.7 % were positive at least for HPV16 and HPV18, respectively. By age group, the prevalence of HPV16/18 was highest for the youngest women in the population at 2.9 % (95 % CI 2.5–3.3) for ages 30–34 and 2.3 % (95 % CI 2.0–2.7) for ages 35–39. Prevalence estimates then declined to 1.8 % (95 % CI 1.5–2.1), 1.3 % (95 % CI 1.0–1.6), 1.4 % (95 % CI 1.0–1.8), and 1.5 % (95 % CI 1.1–2.2) for ages 40–44, 45–49, 50–54, and 55–59 (Table 2 and Fig. 2). Estimates then surge for women aged 60 to 64 to 2.2 % (95 % CI 1.5–3.3) (Table 2 and Fig. 2). The prevalence of HPV16 and HPV18 coinfection but neither hrHPV types was rare (0.08 %) (data not shown). The distribution by age group for women who only had HPV16/18 infections showed a marked U-shaped curve with an initial increase for women in their 30s, a decreased prevalence for women in their 40s and 50s, and a second increase in prevalence for women in their 60s (Table 2 and Fig. 3). Like the other prevalence curves, the coefficients on age^2^ for HPV16/18 only infections is highly significant (Table 3).

Finally, the prevalence of coinfections with HPV16/18 and another hrHPV type was 0.7 %. The prevalence by age showed a similar U-shaped curve with increased estimates for the younger and older women in the population (Table 2 and Fig. 3). Again, this curve had a significant quadratic trend (Table 3). Overall, there was no significant difference between the quadratic terms for HPV16/18 and the non-16/18 hrHPV only curve. Furthermore, when the quadratic terms for HPV16/18 only and non-16/18 hrHPV only were compared, there was no significant difference (*p* < 0.05) (Table 3).

## Discussion

Our population-wide investigation provides a better representation of exposure to infection than a clinic-based population with abnormal cytology in whom HPV16 and HPV18 would be over-represented. The present study is not only the largest in Latin America and most current population based study to date, but is also the first to use Roche’s cobas® 4800 system for hrHPV analysis. Roche’s cobas® 4800 system has been shown to be a highly sensitive and specific test for the detection of hrHPV as well as HPV16 and HPV18 [20].

There has only been one other previous large population based study conducted in Mexico to report hrHPV type specific prevalence estimates. Although this previous study used a different design and methodology, the age distribution of hrHPV from this study shows a similar U-shaped curve with a first peak for the youngest women and a second peak for the oldest age group [11]. A similar U-shaped curve was found in both the Guanacaste cohort in Costa Rica [21] and a population of Colombian women [22]. Outside of Latin America, a meta-analysis of worldwide epidemiologic data also found this same U-shaped curve for developing nations throughout the world including Africa and Southeast Asia [23].

While the second peak of HPV infections for women up to ages 30 to 39 has been seen in diverse populations throughout the world [23, 24], in Central and South America this peak appears to be more prominent. Epidemiologic data suggests that this first peak of infection can be correlated with sexual initiation in younger women when they are exposed to HPV for the first time and their adaptive immune response has yet to develop [8].

The second peak of infections for women ages 60 to 64 in the FRIDA population is again only seen mirrored in populations throughout the developing world. In North America and throughout most of Europe, the prevalence of HPV infection declines consistently after the initial peak associated with sexual debut [23]. The reasons for this second peak of hrHPV DNA detection are unknown. Current theories suggest that this second peak may be the result of immune depression leading to reactivation of previous quiescent infections [25, 26], the acquisition of new hrHPV infections through changes in sexual behavior on the part of the women themselves and/or their partners, or a cohort effect [25, 27–29]. Another theory suggests that differences in screening programs may also play a role in the presence of this second peak. In countries with effective screening programs, the removal of precancerous lesions is believed to have an antigen-presenting effect to the immune system that may help protect against future HPV infections [30]. Based on this theory, in countries where screening programs do not identify and treat these precancerous lesions, women cannot benefit from this added immunologic boost.

For the FRIDA population, the U-shaped prevalence curve was also seen for HPV16/18 infections. This same pattern is seen in the Guanacaste cohort in Costa Rica [21] and in the previous Mexican study for HPV16, but not for HPV18 [11]. Overall, point estimates of HPV16/18 prevalence appear higher in other study populations throughout Latin America [9, 21, 23, 31], but due to different study designs, it is difficult to draw conclusions concerning the comparative burden of infections.

In addition to the differences seen among age groups, multiple sexual partners was found to be a risk factor for hrHPV infection. Other studies have found a similar association between an increased number of sexual partners and risk of hrHPV infection [26, 32, 33]. In contrast, age at first intercourse was not found to be an independent risk factor for hrHPV in this population, similar to other studies. However, we also acknowledge that there are previous studies reporting that the age of sexual debut is an independent risk factor by itself [34]. It should be noted that a particularity of FRIDA population was a younger age of sexual debut was highly correlated with having more than one lifetime sexual partner (*p* < 0.001) (data not shown).

This preliminary analysis only contains 30 % of the target population. With a larger population, our study will be better powered to describe HPV16/18 determinants. We furthermore acknowledge the possibility of social desirability bias with the sensitive, self-reported data, such a bias may have led to underestimation of certain risk factors. We attempted to limit such bias by providing a private space for the recruitment interviews.

In the absence of cervical screening or cancer registries in Mexico at the national, regional or state levels, these prevalence data can help to better inform resource allocation decisions. Sharma et al. developed a model whereby hrHPV prevalence data can be used to estimate cervical cancer risk [5]. While Sharma et al.’s models have been developed to predict cervical cancer incidence in developed countries, more data is needed in developing countries such as Mexico to refine these models so they can be better applied in the developing world [5]. Such a reliable model to determine cervical cancer risk would be a very valuable tool in Mexico and through the developing world. Nonetheless, it is important to note that the generalizability of the findings may be limited both within Mexico and outside of Mexico.

Beyond predicting cervical cancer risk, the prevalence data from this study can serve a number of additional uses from a policy perspective. First the prevalence information can be useful for evaluating the impact of HPV vaccination over the long term. While a number of surrogate endpoints of vaccine impact may be used, HPV type-specific prevalence data has become the standard early indicator of choice [35, 36], and thus these prevalence data may also be used as baseline to monitor vaccine effectiveness.

This prevalence information will furthermore be useful for policymakers trying to determine the best triage practices in Mexico. In 2013, our study group was able to show that primary screening for hrHPV is effective, but a secondary follow-up or triage test is needed to determine which women should be sent to colposcopy [7]. We attempted to send all hrHPV positive women to colposcopy, but found it was not only inefficient, but also overburdened the local health services. In this previous pilot, we sent nearly 11 % of women for colposcopy services. If we had triaged women based on HPV16/18, we would have only sent 2 % of women and still detected more than 70 % of disease [4]. Furthermore, some analyses suggest that the best alternative may be a combination of triage alternatives to offer high joint specificity to detect CIN2 or more, including other triage tests such as cytology, p16 or DNA methylation or at least evidence of 6 to 12 months of persistent hrHPV infection before referral [37]. A triage algorithm using HPV16/18 testing has been validated in the US [10], but needs to be further explored in Mexico. This prevalence data will help policymakers to determine whether or not the current infrastructure in Mexico can support the triage of HPV16 and HPV18 positive women. Further study is still needed to determine the performance and cost-effectiveness of HPV16 and HPV18 detection as an additional triage alternative for hrHPV positive women, and what additional testing may be required to ensure a more effective and safe cervical cancer screening program in Mexico.

The main strength of our study is our large, population-based approach. This is the first study in Mexico of this size to assess the prevalence of HPV16/18. This epidemiologic data will contribute to the creation of a more reliable strategy of cervical cancer prevention in Mexico.

## Conclusion

This sub-analysis of the FRIDA study demonstrates the population level prevalence pattern of hrHPV and HPV16/18 by age in Mexico. Both hrHPV and HPV16/18 positivity have a bimodal distribution by age with higher prevalences at ages 30–39 and 60–64. Infections with hrHPV were also found to be positively associated with an increased number of lifetime sexual partners, a history of STIs, being unmarried, use of hormonal contraception, and reported condom use. Our results set the stage for an improved cervical cancer screening strategy in Mexico. While primary screening for hrHPV has already been instituted in Mexico, this prevalence data suggests the feasibility of HPV typing as an additional screening parameter nationwide.

## Abbreviations

CI: Confidence intervals
CIN: Cervical intraepithelial neoplasia
FRIDA: Forwarding research for improved detection and access
HPV: Human papillomavirus
hrHPV: high-risk human papillomavirus
IUD: Intrauterine device
ORs: Odds ratios
STIs: Sexually transmitted infections

## References

[1] Bruni L, Barrionuevo-Rosas L, Albero G, Aldea M, Serrano B, Valencia S, Brotons M, Mena M, Cosano R, Muñoz J, Bosch FX, de Sanjosé S, Castellsagué X. ICO Information Centre on HPV and Cancer (HPV Information Centre). Human Papillomavirus and Related Diseases in Mexico. Summary Report 2016-02-26. http://www.hpvcentre.net/statistics/reports/MEX.pdf. [Accesed 22 Aug 2016].

[2] Walboomers JMM, Jacobs MV, Manos MM, Bosch FX, Kummer JA, Shah KV, Snijders PJF, Peto J, Meijer CJLM, Muñoz N (1999). Human Papillomavirus is a necessary cause of invasive cervical cancer worldwide. J Pathol.

[3] Muñoz N, Mendez F, Posso H, Molano M, van de Brule AJC, Ronderos M, Meijer CJLM, Muñoz A (2004). Incidence, duration, and determinants of cervical human papillomavirus infection in a cohort of Colombian women with normal cytological results. J Infect Dis.

[4] de Sanjose S, Quint WG, Alemany L, Geraets DT, Klaustermeier JE, Lloveras B, Tous S, Felix A, Bravo LE, Shin H-R, Vallejos CS, de Ruiz PA, Lima MA, Guimera N, Clavero O, Alejo M, Llombart-Bosch A, Cheng-Yang C, Tatti SA, Kasamatsu E, Iljazovic E, Odida M, Prado R, Seoud M, Grce M, Usubutun A, Jain A, Suarez GA, Lombardi LE, Banjo A (2010). Human papillomavirus genotype attribution in invasive cervical cancer: a retrospective cross-sectional worldwide study. Lancet Oncol.

[5] Sharma M, Bruni L, Diaz M, Castellsagué X, de Sanjosé S, Bosch FX, Kim JJ (2012). Using HPV prevalence to predict cervical cancer incidence. Int J Cancer.

[6] Kably-Ambe A, Ruiz-Moreno JA, Lazcano-Ponce E, Vargas-Hernandez VM, Aguado-Perez RA, Alonso-de Ruiz P (2011). Consenso para la prevención del cáncer cervicouterino en México. Ginecol Obstet Mex.

[7] Lazcano-Ponce E, Lorincz AT, Torres L, JJorge S, Cruz A, Rojas R, Hernandez P, Hernandez M (2013). Specimen self-collection and HPV DNA screening in a pilot study of 100,242 women. Int J Cancer.

[8] Moscicki A-B, Shiboski S, Broering J, Powell K, Clayton L, Jay N, Darragh TM, Brescia R, Kanowitz S, Miller SB, Stone J, Hanson E (1998). The natural history of human papillomavirus infection as measured by repeated DNA testing in adolescent and young women. J Pediatr.

[9] Lazcano-Ponce E, Lorincz AT, JJorge S, Fernández I, Cruz A, Hernandez P, Mejia I, Hernández-Ávila M (2010). A pilot study of HPV DNA and cytology testing in 50,159 women in the routine Mexican Social Security Program. Cancer Causes Control.

[10] Cox JT, Castle PE, Behrens CM, Sharma A, Wright TC, Cuzick J, Group AHS (2013). Comparison of cervical cancer screening strategies incorporating different combinations of cytology, HPV testing, and genotyping for HPV 16/18:results from the ATHENA HPV study. Ame J Obstet Gynecol.

[11] Lazcano-Ponce E, Herrero R, Muñoz N, Cruz A, Shar KV, Alonso P, Hernandez P, JJorge S, Hernandez M (2001). Epidemiology of HPV infection among Mexican women with normal cervical cytology. Int J Cancer.

[12] Orozco-Colin A, Carrillo-García A, Méndez-Tenorio A, Ponce-de-León S, Mohar A, Maldonado-Rodríguez R, Guerra-Arias R, Flores-Gil O, Sotelo-Regil R, Lizano M (2010). Geographical variation in human papillomavirus prevalence in Mexican women with normal cytology. Int J Infect Dis.

[13] Velázquez-Márquez N, Paredes-Tello MA, Pérez-Terrón H, Santos-López G, Reyes-Leyva J, Vallejo-Ruiz V (2009). Prevalence of human papillomavirus genotypes in women from a rural region of Puebla, Mexico. Int J Infect Dis.

[14] Sánchez-Anguiano L, Alvarado-Esquivel C, Reyes-Romero M, Carrera-Rodríguez M (2006). Human papillomavirus infection in women seeking cervical Papanicolaou cytology of Durango, Mexico: prevalence and genotypes. BMC Infect Dis.

[15] Illades-Aguiar B, Cortés-Malagón E-M, Antonio-Véjar V, Zamudio-López N, Alarcón-Romero LDC, Fernández-Tilapa G, Hernández-Sotelo D, Terán-Porcayo M-A, Flores-Alfaro E, Leyva-Vázquez M-A (2009). Cervical carcinoma in southern Mexico: human papillomavirus and cofactors. Cancer Detect Prev.

[16] Illades-Aguiar B, del Carmen Alarcón-Romero L, Antonio-Véjar V, Zamudio-López N, Sales-Linares N, Flores-Alfaro E, Fernández-Tilapa G, Vences-Velázquez A, Muñoz-Valle JF, Leyva-Vázquez M-A (2010). Prevalence and distribution of human papillomavirus types in cervical cancer, squamous intraepithelial lesions, and with no intraepithelial lesions in women from Southern Mexico. Gynecol Oncol.

[17] Peralta-Rodriguez R, Romero-Morelos P, Villegas-Ruiz V, Mendoza-Rodriguez M, Taniguchi-Ponciano K, Gonzalez-Yebra B, Marrero-Rodriguez D, Salcedo M (2012). Prevalence of human papillomavirus in the cervical epithelium of Mexican women: meta-analysis. Infect Agents Cancer.

[18] Torroella-Kouri M, Morsberger S, Carrillo A, Mohar A, Meneses A, Ibarra M, Daniel RW, Ghaffari AM, Solorza G, Shah KV (1998). HPV prevalence among Mexican women with neoplastic and normal cervixes. Gynecol Oncol.

[19] Snijders PJF, Heideman DAM, Meijer CJLM (2010). Methods for HPV detection in exfoliated cell and tissue specimens. APMIS.

[20] Park Y, Lee E, Choi J, Jeong S, Kim HS (2012). Comparison of the Abbott RealTime high-risk human papillomavirus (HPV), roche cobas HPV, and hybrid capture 2 assays to direct sequencing and genotyping of HPV DNA. J Clin Microbiol.

[21] Herrero R, Castle PE, Schiffman M, Bratti C, Hildesheim A, Morales J, Alfaro M, Sherman ME, Wacholder S, Chen S, Rodriguez AC, Burk RD (2005). Epidemiologic profile of type-specific human papillomavirus infection and cervical neoplasia in Guanacaste, costa rica. J Infect Dis.

[22] Molano M, Posso H, Weiderpass E, van den Brule AJC, Ronderos M, Franceschi S, Meijer CJLM, Arslan A, Munoz N (2002). Prevalence and determinants of HPV infection among Colombian women with normal cytology. Br J Cancer.

[23] Bruni L, Diaz M, Castellsagué X, Ferrer E, Bosch FX, de Sanjose S (2010). Cervical human papillomavirus prevalence in 5 continents: MetahAnalysis of 1 million women with normal cytological findings. J Infect Dis.

[24] Franceschi S, Herrero R, Clifford GM, Snijders PJF, Arslan A, Anh PTH, Bosch FX, Ferreccio C, Hieu NT, Lazcano-Ponce E, Matos E, Molano M, Qiao Y-L, Rajkumar R, Ronco G, de Sanjose S, Shin H-R, Sukvirach S, Thomas JO, Meijer CJLM, Muñoz N, the IARC HPV Prevalence Surveys Study Group (2006). Variations in the age-specific curves of human papillomavirus prevalence in women worldwide. Int J Cancer.

[25] Gravitt PE (2012). Evidence and impact of human papillomavirus latency. Open Virol J.

[26] Rositch AF, Burke AE, Viscidi RP, Silver MI, Chang K, Gravitt PE (2012). Contributions of recent and past sexual partnerships on incident human papillomavirus detection: acquisition and reactivation in older women. Cancer Res.

[27] Bosch FX, Burchell AN, Schiffman M, Giuliano AR, de Sanjose S, Bruni L, Tortolero-Luna G, Kjaer SK, Muñoz N (2008). Epidemiology and natural history of human papillomavirus infections and type-specific implications in cervical neoplasia. Vaccine.

[28] Trottier H, Ferreira S, Thomann P, Costa MC, Sobrinho JS, Prado JCM, Rohan TE, Villa LL, Franco EL (2010). Human papillomavirus infection and reinfection in adult women: the role of sexual activity and natural immunity. Cancer Res.

[29] Gravitt PE, Rositch AF, Silver MI, Marks MA, Chang K, Burke AE, Viscidi RP (2013). A cohort effect of the sexual revolution May Be masking an increase in human papillomavirus detection at menopause in the United States. J Infect Dis.

[30] Passmore J-AS, Morroni C, Shapiro S, Williamson A-L, Hoffman M (2007). Papanicolaou smears and cervical inflammatory cytokine responses. J Inflamm.

[31] Matos E, Loria D, Amestoy GM, Herrera L, Prince MA, Moreno J, Krunfly C, van den Brule AJC, Meijer CJLM, Muñoz N, Herrero R (2003). Prevalence of human papillomavirus infection among women in Concordia. Argentina: Sex Transm Dis.

[32] Bahmanyar ER, Paavonen J, Naud P, JJorge S, Chow S-N, Apter D, Kitchener H, Castellsagué X, Teixeira JC, Skinner SR, Jaisamrarn U, Limson GA, Garland SM, Szarewski A, Romanowski B, Aoki F, Schwarz TF, Poppe WAJ, De Carvalho NS, Harper DM, Bosch FX, Raillard A, Descamps D, Struyf F, Lehtinen M, Dubin G (2012). Group FTHPS: prevalence and risk factors for cervical HPV infection and abnormalities in young adult women at enrolment in the multinational PATRICIA trial. Gynecol Oncol.

[33] Roura E, Iftner T, Vidart JA, Kjaer SK, Bosch FX, Muñoz N, Palacios S, Rodriguez MSM, Morillo C, Serradell L, Torcel-Pagnon L, Cortes J, Castellsagué X (2012). Predictors of human papillomavirus infection in women undergoing routine cervical cancer screening in Spain: the CLEOPATRE study. BMC Infect Dis.

[34] Kahn JA, Rosenthal SL, Succop PA, Ho GYF, Burk RD (2002). Mediators of the association between Age of first sexual intercourse and subsequent human papillomavirus infection. Pediatrics.

[35] Wheeler CM, Hunt WC, Cuzick J, Langsfeld E, Pearse A, Montoya GD, Robertson M, Shearman CA, Castle PE (2012). For the New Mexico HPV Pap registry steering committee: a population-based study of human papillomavirus genotype prevalence in the United States: baseline measures prior to mass human papillomavirus vaccination. Int J Cancer.

[36] Chang Y, Brewer NT, Rinas AC, Schmitt K, Smith JS (2009). Evaluating the impact of human papillomavirus vaccines. Vaccine.

[37] Beal CM, Salmerón J, Flores YN, Torres L, Granados-García V, Dugan E, Lazcano-Ponce E (2014). Cost analysis of different cervical cancer screening strategies in Mexico. Salud Publica Mex.

